# Effect of curing ingredients and vacuum packaging on the physico-chemical and storage quality of ready-to-eat *Vawksa rep* (smoked pork product) during refrigerated storage

**DOI:** 10.14202/vetworld.2016.587-594

**Published:** 2016-06-12

**Authors:** Deepshikha Deuri, Pragati Hazarika, Tarun Pal Singh, Lalchamliani Chhangte, Parminder Singh, Suman Talukder

**Affiliations:** 1 Division of Livestock Products Technology, College of Veterinary Sciences and Animal Husbandry, CAU, Selesih, Aizawl, Mizoram, India; 2 Division of Livestock Products Technology, Indian Veterinary Research Institute, Izzatnagar, Bareilly, Uttar Pradesh, India; 3 Veterinary Officer, Department of Animal Husbandry, Government of Punjab, Amritsar, Punjab, India

**Keywords:** curing, smoking, storage quality, vacuum packaging, *Vawksa rep*

## Abstract

**Aim:**

The present study was conducted for the development of ready-to-eat *Vawksa rep* (smoked pork product) and to study the synergistic effect of curing ingredients and vacuum packaging on the physico-chemical and storage quality during refrigerated storage at (4°C±1°C) for 15 days.

**Materials and Methods:**

Four different batches of *Vawksa rep* samples were prepared, i.e., T-1 (uncured, first cooked at 121°C for 15 min, and then smoked at 120°C for 30 min), T-2 (uncured, cooked, and smoked simultaneously at 120°C for 45 min), T-3 (cured, first cooked at 121°C for 15 min, and then smoked at 120°C for 30 min), and T-4 (cured, cooked, and smoked simultaneously at 120°C for 45 min).

**Results:**

Cooking yield was significantly higher (p<0.05) for the T-4. The pH of T-3 and T-4 samples was significantly higher (p<0.05) on day 15. The tyrosine value of all the samples increased significantly (p<0.05) among the different days of analysis. Thiobarbituric acid value was significantly (p<0.05) lower in T-3 sample both at the beginning and at the end of storage period. In microbiological profile, total plate count was lower in T-3 and T-4 than T-1 and T-2. However, *Escherichia coli* count was negative for T-3 and T-4 samples throughout the storage period. Among sensory attributes, T-3 and T-4 samples registered superior scores for color, flavor, texture, juiciness, and overall acceptability.

**Conclusion:**

Furthermore, *Vawksa rep* (smoked pork product) could be prepared easily with little technology up-gradation and with a negligible escalation of production cost.

## Introduction

Meat is a highly perishable commodity due to a rich source of nutrients. Color, microbial growth, and lipid oxidation are considered important factors for limiting the shelf-life and consumer acceptance of meat and meat products [1]. Thus for extending the shelf-life, the use of a combination technology of different processing, packaging, and preservation conditions is a need of the hour.

Smoking is one of the oldest food processing technologies which not only improve the sensory quality but also inactivates a number of enzymes and microflora [2]. The active components of smoking which produce these activities are phenols, carbonyls, acids, alcohols, esters, lactones, polycyclic aromatic hydrocarbons, pyrazines, pyrrole, and furan derivatives [3]. It mainly improves color, odor and texture (firmness/hardness), physico-chemical, and microbial quality. However, smoking is often coupled with curing, salting, packaging, and chilling techniques to produce synergistic effects toward spoilage micro-organisms and to increase the shelf-life [4]. Vacuum packaging of meat is meant to retard or completely check the oxidative reactions and inhibit the microbial growth by reducing the amount of oxygen in contact with the product. Vacuum packaging preserves the natural flavor of the product, in addition to a number of advantages including such as saving of space and energy during storage, transport and distribution, reduction in weight loss, better-keeping qualities, and better display of the product [5].

Ready-to-eat food products form an important part of the diet in our day-to-day life. *Vawksa rep*, not ready-to-eat but a ready-to-cook smoked pork product, is one of the most popular traditional meat products of Mizoram. *“Sa-rep”* means “smoked meat” (where “*Sa”* stands for meat and “*rep”* means dehydrated or dry ones in Mizo language) is prepared locally in Mizoram and is preferred and widely consumed by the majority of middle-class income Mizo people [6]. It is prepared by mild smoking (2-3 h) of pork chunks of almost uniform size. The problem associated with *Vawksa rep* is that it has a short shelf-life because pork is highly prone to lipid oxidation due to the presence of excessive amounts of fat in it, and second, no other processing and preservation (except smoking) treatments are applied to this product for prolonging its shelf-life. Moreover, the product is sold without proper packaging, thereby increasing the chances of physical, chemical, and microbial contamination, in addition to fat oxidation. Keeping in view of the above-mentioned problems, the present study was undertaken for the development of ready-to-eat *Vawksa rep* and to study the synergistic effect of curing, smoking, and vacuum packaging on the physico-chemical, sensory, and microbial quality during refrigerated storage conditions.

## Materials and Methods

### Preparation of Vawksa rep meat samples

Raw pork cuts (hams) were purchased from the freshly slaughtered Yorkshire pig carcasses of about 8 months to 1 year of age from local market. In the laboratory, these were cut into small pieces of uniform size (3″×3″) with the help of a knife. Traditional *Vawksa rep* was prepared by piercing the pork chunks to the wooden stick and then placing the meat chunks 30 cm above the fire for 45 min. Hardwood was used for smoking of meat. Uncured pork chunks were first pressure cooked at 121°C for 15 min and subsequently smoked (CS) in the smoke unit (Kerres Showsmoker CS 350 EL) for 30 min at 120°C (T-1); cooked and smoked simultaneously (Directly smoked, DS) in the smoke unit at 120°C for 60 min (T-2). The pork chunks of uniform sizes (3″×3″) were cured for 24 h using curing mixture (Table-1). Cured pork chunks were pressure cooked at 121°C for 15 min and subsequently smoked in the smoke unit for 30 min at 120°C (T-3); cooked and smoked simultaneously in the smoke unit at 120°C for 45 min (T-4). These treatments were packed and sealed using the vacuum packaging machine in high-density polyethylene (HDPE, 3 Mil) bags and kept at refrigeration temperature (4°C±1°C) for 15 days. The samples were analyzed for different physico-chemical, sensory, and microbiological quality parameters at 1, 5, 10, and 15 days.

**Table-1:** Formulation for marination of pork chunks.

Ingredients	Quantity
Salt	1.5%
Sodium nitrate (NaNO_3_)	200 mg/kg
Sodium nitrite (NaNO_2_)	150 mg/kg
Spice-mix	2%
Sodium tripolyphosphate	0.5%
Yoghurt	10%
Sugar	1.5%

### Physico-chemical quality parameters

#### Cooking yield

Cooking yield was calculated by dividing the weights of raw and cooked sample before with that of after cooking, multiplied by 100.

#### Cooking loss

Cooking loss was calculated by subtracting weight of the cooked product from the weight of the raw pork chunks.

#### pH

The pH of the sample was determined using a pH meter (Cyberscan 1000 Euteoh Instruments) and by following the methods as described by Bendall [7].

#### Texture analysis

Texture of the samples was analyzed using food texture analyzer (TA HD- Plus) with a load cell of 250 kg on different days of analysis. Measurements were taken at room temperature with a sample size of 2×2 cm^3^. Data collection and analysis were performed using the Texture Expert Stable Micro Systems computer program, version 1.16.

#### Tyrosine value (TV) and thiobarbituric acid value (TBA)

TV and TBA were determined as per the standard methods proposed by Pearson [8] and Witte *et al*. [9], respectively.

### Microbiological quality parameters

Total plate count (TPC), coli titer count, and *E. coli* counts of the samples were performed following the methods as described by APHA [10]. The average number of colonies was multiplied by the reciprocal of the dilution and expressed as log_10_ cfu/g.

### Organoleptic quality parameters

#### Assessment of odor

All the *Vawksa rep* meat samples were assessed by a semi-trained panel of 25-30 judges by using a 10 point hedonic scorecard scale [8] on day 1^st^ and thereafter, on 5^th^, 10^th^, and 15^th^ days of storage.

#### Sensory evaluation

A six member experienced panel of judges evaluated the samples for different sensory attributes, viz., color and appearance, flavor, texture, juiciness, and overall acceptability, using an 8-point descriptive scale [11], where 8=excellent and 1=extremely poor. On each day of analysis, samples were cooked at 121°C for 15 min, cooled and then presented to the panelists for sensory evaluation along with a glass of water for rinsing of mouth.

### Statistical analysis

Data were analyzed statistically on “SPSS-16.0” (SPSS Inc., Chicago, II USA) software package as per standard methods [12]. Duplicate samples were drawn for each parameter, and the whole experiment was repeated three times to have total number of observations, n=6 for all parameters. The entire data were subjected to two-way analysis of variance along with Duncan’s multiple range test, and the significance was studied at 5% level (p<0.05).

## Results and Discussion

### Physico-chemical quality

Cooking yields and cooking losses were estimated at day 0. Statistical analysis revealed that cooking yield was significantly higher (p<0.05) for the T-4 (84.61±0.02^d^%) followed by T-2 (82.63±0.09^c^%), T-3 (82.50±0.05^b^%), and T-1 (81.75±0.05^a^%). It might be due to the extra uptake of salt and sugar, which were retained during processing, thus increasing water retention in T-4. The cooking loss was significantly higher (p<0.05) for T-1 (18.25±0.03^d^%) followed by T-3 (17.50±0.15^c^%), T-2 (17.37±0.03^b^%), and T-4 (15.39±0.09^a^%). The heat treatment causes more moisture losses from the product, thereby resulting into more loss of meat fluid in T-1. Kanithaporn *et al*. [13] observed that the cooking loss in fully cooked RTE bacon heated for 30 s (20.91±0.68) was lower than that of the RTE bacon heated for 60 s (24.23±0.32).

The results for pH, texture analysis (hardness), TV, and TBA are presented in Table-2. The mean initial pH value of raw pork was 6.55. Initially, the pH values decreased up to 5^th^ day of storage in all the treatment groups, but thereafter a rise in pH was observed gradually up to 15^th^ day of storage, except in T-3, where progressive decrease in pH was recorded on 1^st^, 5^th^, and 10^th^ days of storage followed by increased pH on 15^th^ day of storage. CS samples, i.e., T-1 and T-3 for producing RTE *Vawksa rep* exhibited higher pH values compared to the ones who were directly smoked, i.e., T-2 and T-4. The pH of T-3 and T-4 samples was significantly higher (p<0.05) on day 15 than day 1. Overall DS samples showed a faster fall in pH than CS samples of RTE *Vawksa rep*. Arnim and Marlida [14] also reported increase in pH values (at the end of storage) of meat balls treated with liquid smoke. Similar findings were observed by Kumar *et al*. [15] in pork nuggets during their refrigerated storage. The increase in pH of T-1, T-2, and T-4 on 10^th^ and 15^th^ day of storage, respectively, was in agreement with the findings of Karabagias *et al*. [16]. The variation in pH with curing ingredients, among the different treatments could be attributed to various factors such as the formation of bacterial metabolites, deamination of proteins, growth of facultative anaerobes, lactic acid bacteria, migration of antimicrobial substances, and formation of carbonic acid in vacuum packaging [17].

**Table-2:** Physico-chemical properties of *Vawksa* *rep* (smoked pork product) stored under vacuum packaging conditions at 4°C±1°C.

Treatments/Days	1	5	10	15
pH				
T-1	6.50±0.01^aA^	6.45±0.01^aA^	6.54±0.13^aA^	6.60±0.01^aA^
T-2	6.44±0.01^aAB^	6.25±0.01^cC^	6.37±0.01^bAB^	6.45±0.01^aB^
T-3	6.46±0.02^aB^	6.38±0.01^bcB^	6.36±0.01^cAB^	6.40±0.01^bC^
T-4	6.43±0.01^aB^	6.24±0.01^dC^	6.30±0.01^cB^	6.39±0.01^bC^
Hardness (g)				
T-1	2287.21±0.06^dA^	2687.80±0.06^cA^	2785.80±0.10^bA^	2869.17±0.32^aA^
T-2	1890.41±0.06^dB^	2412.90±0.10^cB^	2577.60±0.06^bB^	2683.60±0.06^aB^
T-3	1775.50±0.06^dC^	1832.90±0.10^cC^	1960.92±0.11^bC^	2041.70±0.06^aC^
T-4	1576.60±0.10^dD^	1650.30±0.10^cD^	1681.40±0.10^bD^	1712.50±0.14^aD^
TV (mg tyrosine/100 g)				
T-1	0.058±0.05^dB^	0.075±0.05^cB^	0.089±0.01^bB^	0.150±0.01^aB^
T-2	0.085±0.08^dA^	0.101±0.01^cA^	0.152±0.06^bA^	0.165±0.09^aA^
T-3	0.052±0.06^cC^	0.055±0.06^cD^	0.060±0.01^bC^	0.098±0.09^aD^
T-4	0.059±0.06^dB^	0.065±0.09^cC^	0.087±0.05^bB^	0.103±0.01^aC^
TBA (mg malondialdehyde/kg)				
T-1	0.28±0.05^dA^	0.34±0.09^cA^	0.65±0.08^bC^	1.21±0.08^aB^
T-2	0.25±0.01^dA^	0.32±0.05^cAB^	0.80±0.09^bA^	1.35±0.01^aA^
T-3	0.21±0.08^dB^	0.30±0.05^cB^	0.75±0.01^bB^	0.80±0.09^aD^
T-4	0.25±0.01^dA^	0.32±0.05^cAB^	0.78±0.05^bA^	0.90±0.09^aC^

n=6, mean±SE with different superscripts row wise (small alphabets) and column wise (capital alphabets) differ significantly (p<0.05). SE=Standard error, TV=Tyrosine value, TBA=Thiobarbituric acid

In texture profile, with the advancement of the storage period, there was continued and significant (p<0.05) increase in hardness of all the *Vawksa rep* samples. In general, higher mean peak forces were required to cut CS as compared to DS RTE *Vawksa rep* samples. Between CS, i.e. T-1 and T-3; the T-3 (1775.50-2041.70 g) required low force than T-1 (2287.21-2869.17 g). In DS *RTE Vawksa rep* (T-2 and T-4), a relatively lower force was required in T-4 (1576.60-1712.50 g) as compared to T-2 (1890.41-2683.60 g). Garcia-Esteban *et al*. [18] also observed continued increase in hardness value of dry cured hams during storage under vacuum packaging conditions, with the highest hardness value (2669.00 g) observed in vacuum packed samples at the end of storage period. Cilla *et al*. [19] also observed a hardness value of 5220.99 g in vacuum packaged dry cured ham cuts stored at 4°C±2°C.

The mean initial TV of raw pork was 0.220 mg tyrosine/100 g (0 day). Irrespective of the treatment groups, the TV increased in all the samples throughout the storage period (Table-2). The mean TV of all the samples increased significantly (p<0.05) among the different days of analysis. At the end of the storage period, i.e., day 15, TV was significantly higher (p<0.05) in T-2 followed by T-1, T-4, and T-3. Even though all the treatment groups showed a gradual increase in the TV from 1^st^ to 15^th^ day, the DS RTE *Vawksa rep* exhibited higher TV than the CS RTE *Vawksa rep* samples. Similar results were reported by Lalchamliani *et al*. [20] in *Vawksa rep* samples stored under different aerobic vacuum packaging conditions at 4°C±1°C. The findings are also in agreement with the statement of Pearson [8], who reported that TV of meat increases with storage period until deamination of amino acid limits the formation of free amino acid.

The TBA value of raw pork was 0.235 mg malondialdehyde/kg of meat. In general, TBA values of all the samples increased significantly (p<0.05) throughout the storage period (Table-2). Compared to other treatments, T-3 sample maintained significantly (p<0.05) lower values of TBA both at the beginning (day 1) and at the end (day 15) of storage period. At the end of the storage period, i.e., on day 15, the difference in TBA values of both CS and DS RTE *Vawksa rep* samples was significantly higher (p<0.05) than day 1, 5 and 10 of storage periods. For TBA value, 0.5 mg malondialdehyde/kg is considered as the cut-off value for fat oxidation and 1 mg malondialdehyde/kg for rancidity in the products. From the Table-2, it is clear that all the treatments of RTE *Vawksa rep* experienced oxidation on the 10^th^ day of storage. Overall, the combination of smoking and vacuum packaging resisted the lipid oxidation in RTE *Vawksa rep*. In this study, the increase in TBA values of all the samples was within the threshold limit of 1-2 mg malondialdehyde/kg meat. The increase in TBA value during storage might be attributed to the oxidation of meat lipid or both CS and DS RTE *Vawksa rep* at refrigeration temperature. Lalchamliani *et al*. [20] also observed increasing trends in TBA values of *Vawksa rep* samples during refrigerated storage. Kumar *et al*. [15] also reported that the TBARS values of pork nuggets significantly (p<0.01) increased with the increase of storage period. Kumar and Sharma [21] also observed a linear increase in TBA value throughout the storage period while studying the quality characteristics of low-fat ground pork patties containing milk co-precipitate.

### Microbiological quality

The data related to microbiological quality parameters of *Vawksa rep* are presented in Table-3. The TPC in raw pork was 5.50 log_10_ cfu/g. There was a continued and significant increase (p<0.05) in TPC of all the samples from day 1 to 15, though the increase in the count was relatively less in T-3 and T-4 than T-1 and T-2. Moreover, it was evidenced that smoking caused decrease in the number of the viable aerobic micro-organisms from the initial mean value of raw meat. The increase in the TPC *Vawksa rep* during storage might be due to increasing in pH values of the sample, which favored the growth of bacteria. The reduction in the count of aerobic microbes could also be due to the antimicrobial effect of curing ingredients, smoking, and vacuum packaging. The findings are in agreement with the results of Lalchamliani *et al*. [20] in *Vawksa rep* meat samples stored at 4°C±1°C. Irkin *et al*. [22] also observed that minced beef samples treated with vacuum packaging showed lower viable counts than control. Kumar *et al*. [15] reported gradual but significant increase in total viable counts throughout the storage period in pork nuggets. Lawrie [23] reported that when smoking was combined with curing, the shelf-life of such products increases and the microbial load decreases especially on the meat surface.

**Table-3:** Microbiological quality of *Vawksa* *rep* (smoked pork product) stored under vacuum packaging conditions at 4°C±1°C.

Treatments/Days	1	5	10	15
Total plate count (log_10_ cfu/g)				
T-1	3.60±0.06^dA^	3.90±0.06^cA^	4.80±0.04^bA^	5.30±0.06^aA^
T-2	3.80±0.10^dA^	4.10±0.06^cA^	4.90±0.06^bA^	5.40±0.10^aA^
T-3	2.51±0.12^bB^	2.60±0.10^bB^	3.50±0.14^aB^	3.80±0.06^aB^
T-4	2.60±0.10^bB^	2.70±0.06^bB^	3.70±0.13^aB^	3.90±0.06^aB^
Colititer (MPN/g)				
T-1	115.00±11.69^dB^	310.00±52.09^cB^	343.33±55.29^bB^	493.33±129.07^aB^
T-2	134.16±10.83^dA^	353.33±48.83^cA^	386.66±46.38^bA^	530.00±119.53^aA^
T-3	15.00±0.37^bC^	ND	ND	ND
T-4	18.00±1.71^bD^	ND	ND	ND
*E. coli* count (log_10_ cfu/g)				
T-1	1.57±0.05^cA^	1.73±0.05^bA^	1.82±0.09^abA^	1.93±0.05^aA^
T-2	1.62±0.05^dA^	1.75±0.05^cA^	1.85±0.05^bA^	1.98±0.03^aB^
T-3	ND	ND	ND	ND
T-4	ND	ND	ND	ND

n=6, mean±SE with different superscripts row wise (small alphabets) and column wise (capital alphabets) differ significantly (p<0.05). ND=Not detected, SE=Standard error, *E. coli=Escherichia coli*

The mean colititer value of raw pork was 780.00 MPN/g. The mean values of colititer counts increased significantly (p<0.05) in T-1 and T-2. However, in T-3 and T-4 samples, colititer count was detected only on day 1 and was not detected subsequently. Among T-3 and T-4, colititer count was significantly lower (p<0.05) in T-3 sample. Hence, in terms of microbiological quality, T-3 sample of *Vawksa rep* was superior to other variants. Irkin *et al*. [22] reported that the number of coliforms increased slightly in all the minced beef samples until the end of storage and was below the accepted limit (3 log_10_ cfu/g) except in control samples stored at 4°C. Bacteriological isolation studies by Chaudhari *et al*. [6] in Mizoram involving 100 pork “*Sa-rep*” (smoked meat) samples revealed the presence of *E. coli* only in 9 isolates. The initial mean *E. coli* count was recorded to be 3.12 log_10_ cfu/g of meat. There was a significant increase (p<0.05) in *E. coli* counts in T-1 and T-2 from the mean value of 1.57 log_10_ cfu/g and 1.62 log_10_ cfu/g on the 1^st^ day to 1.93 and 1.98 log_10_ cfu/g on the 15^th^ day of storage, respectively. The *E. coli* count was negative for all the cured CS and DS (T-3 and T-4) products in all the storage periods. The absence of *E. coli* in T-3 and T-4 conforms to the national standard, i.e., nil presence of coliform organisms at 0.001g level the for entire storage period.

### Organoleptic quality

#### Assessment of odor

The odor scores for different treatments decreased significantly with the advancement of the storage period (Table-4). In general, the scores were comparatively higher for T-3 and T-4 than T-1 and T-2. At the beginning of storage period, i.e., day 1, the odor scores for T-3 and T-4 were similar (9.60), but at the end of the storage period (day 15), the scores were slightly higher (p<0.05) for T-3 (7.80) than T-4 (7.60). The decrease in odor scores of samples might be due to continue and significant (p<0.05) increase in TBA value throughout the storage period. Many authors reported similar findings and observed that meat products were acceptable and did not show any perceivable rancidity or off odor/aroma up to 21 days of storage [24]. Nathappan *et al*. [25] also reported declining trends in odor scores of stored mutton (5°C±1°C) with the advancement of the storage period, and the scores also remained within the limit of acceptability up to 72 h of storage.

**Table-4:** Odor scores of *Vawksa* *rep* (smoked pork product) stored under vacuum packaging conditions at 4°C±1°C.

Treatments/Days	1	5	10	15
T-1	9.20±0.05^aA^	8.50±0.05^bB^	6.70±0.05^cB^	6.20±0.05^dB^
T-2	9.40±0.05^aA^	8.40±0.05^bB^	6.50±0.05^cB^	6.10±0.09^dB^
T-3	9.60±0.05^aA^	9.50±0.05^abA^	8.30±0.05^cA^	7.80±0.05^dA^
T-4	9.60±0.05^aA^	9.40±0.05^abA^	8.20±0.05^cA^	7.60±0.05^dAB^

n=27, mean±SE with different superscripts row wise (small alphabets) and column wise (capital alphabets) differ significantly (p<0.05). SE=Standard error

#### Sensory evaluation

The results of the sensory evaluation are presented in Table-5. The color and appearance scores were significantly higher (p<0.05) in T-3 and T-4, both at beginning (day 1) and at the end (day 15) of the storage period. However, in general, the scores for color decreased in all the samples as the storage advanced. At the end of storage, the scores for color and appearance were highest for T-3 (7.00) followed by T-4 (6.80), T-2 (5.70), and T-1 (5.50). The results were inconsistent with the findings of Lalchamliani *et al*. [20] in *Vawksa rep* meat samples stored at 4°C±1°C. Under vacuum packaging conditions, meat is protected from color fading due to the low level of oxygen [26]. Both the cured CS and DS (T-3 and T-4) RTE *Vawksa rep* showed superior flavor scores as compared to the uncured CS and DS (T-1 and T-2). The flavor scores also followed a declining trend starting from beginning till the end of the storage period, which might be due to increase in TBA, TPC, and coliform count. Overall the flavor scores were significantly higher (p<0.05) in T-3 and T-4 on all the storage days. Devatkal *et al*. [27] reported that there is deterioration of flavor due to microbial growth and oxidative rancidity in restructured pork rolls. As the storage period is advanced, the sharp decline in flavor scores might be due to oxidation of fat [28], liberation of fatty acids [29] and increased microbial load [30]. Both CS (T-1 and T-3) RTE *Vawksa rep* registered high texture scores as compared to the DS (T-2 and T-4) RTE *Vawksa rep*. Texture also followed declining trend throughout the storage in all the samples. Azad [31] also reported that during storage of smoked buffalo meat at 0°C and −4°C, it started losing color, texture, and odor. On all the storage days, juiciness was found to be significantly higher (p<0.05) in T-3 and T-4 batches than T-1 and T-2. Throughout the storage period, highest juiciness was observed in T-4 among the different samples. Overall, juiciness scores declined progressively from 1^st^ to 15^th^ day of storage, which might be due to significant (p<0.05) increase in hardness values in all the samples. Kumar *et al*. [15] also observed a significant decrease in flavor, texture, and juiciness of pork nuggets during storage. Decrease in texture and juiciness of samples might be due to loss of moisture during the storage. The overall acceptability was significantly higher (p<0.05) in T-3 on all the storage days. Both the cured CS and DS (T-3 and T-4) RTE *Vawksa rep* had registered higher overall acceptability as compared to their uncured counterparts, i.e. CS and DS (T-1 and T-2). In general, overall acceptability decreased in all the samples as the storage period advanced. These findings are in agreement with the report of Iwanegbe *et al*. [32], who also observed significant differences (p<0.05) among the treatments groups (different cures, storage periods, and storage temperatures) in terms of color, flavor, juiciness, tenderness, and overall acceptability of smoked rabbit meat.

**Table-5:** Sensory properties of *Vawksa* rep (smoked pork product) stored under vacuum packaging conditions at 4°C±1°C.

Treatments/Days	1	5	10	15
Color and appearance				
T-1	6.20±0.05^aB^	6.00±0.14^bA^	5.80±0.12^aB^	5.50±0.15^aB^
T-2	6.50±0.09^aB^	6.30±0.05^aB^	6.00±0.14^bB^	5.70±0.08^cB^
T-3	7.50±0.18^aA^	7.30±0.09^abA^	7.20±0.09^abA^	7.00±0.05^bA^
T-4	7.30±0.09^aA^	7.10±0.05^abA^	7.00±0.14^abA^	6.80±0.12^bA^
Flavor				
T-1	6.20±0.05^aB^	6.00±0.05^aB^	5.70±0.05^bB^	5.40±0.09^cB^
T-2	6.00±0.18^aB^	5.80±0.09^aB^	5.40±0.05^bC^	5.20±0.09^bB^
T-3	7.60±0.05^aA^	7.30±0.07^bA^	7.10±0.03^bcA^	7.00±0.14^cA^
T-4	7.40±0.12^aA^	7.20±0.09^abA^	7.00±0.14^bA^	6.90±0.03^bA^
Texture				
T-1	6.60±0.07^aB^	6.50±0.14^aB^	6.00±0.07^bB^	5.80±0.05^bBC^
T-2	6.40±0.05^aB^	6.20±0.07^bC^	5.90±0.05^cBC^	5.70±0.05^dC^
T-3	7.60±0.05^aA^	7.40±0.05^abA^	7.20±0.09^bcA^	7.00±0.13^cA^
T-4	7.40±0.07^aA^	7.30±0.05^aA^	7.10±0.05^bAB^	6.80±0.05^cAB^
Juiciness				
T-1	6.50±0.13^aB^	6.30±0.09^abB^	6.20±0.09^abB^	6.00±0.13^bB^
T-2	6.60±0.03^aB^	6.50±0.05^aB^	6.30±0.05^bB^	6.10±0.09^cB^
T-3	7.40±0.09^aA^	7.20±0.05^aA^	6.90±0.09^bA^	6.70±0.09^bA^
T-4	7.50±0.05^aA^	7.30±0.12^abA^	7.00±0.14^bcA^	6.80±0.13^cA^
Overall acceptability				
T-1	6.50±0.15^aB^	6.30±0.05^abC^	6.00±0.13^bB^	5.50±0.09^cB^
T-2	6.30±0.05^aB^	6.00±0.14^bD^	5.80±0.09^bB^	5.30±0.05^cB^
T-3	7.80±0.05^aA^	7.60±0.09^aA^	7.30±0.02^bA^	7.00±0.13^aA^
T-4	7.50±0.13^aA^	7.30±0.05^abB^	7.10±0.05^bA^	6.80±0.09^cA^

n=18, mean±SE with different superscripts row wise (small alphabets) and column wise (capital alphabets) differ significantly (p<0.05). SE=Standard error

### Production economics of cured versus uncured Vawksa rep

The comparative cost for the formulation of each of 25 kg CS (T-1 and T-3) and DS (T-2 and T-4) is presented in Table-6. It includes the cost of raw materials required, viz., cost of raw pork, curing ingredients (salt, sugar, nitrate, and nitrite), spice mix, yoghurt, STPP, packaging, saw dust/wood, and electricity. The cost of production of cured CS and DS RTE *Vawksa rep* was estimated to be Rs. 269.57/kg, whereas for uncured CS and DS RTE *Vawksa* rep, it was Rs 254.12/kg. The expenditure incurred toward equipment and labor was not considered. The production cost of cured CS and DS RTE *Vawksa rep* was somewhat higher than the uncured CS and DS RTE *Vawksa rep*, but at the same time, it is much lower than the present market price of *Vawksa rep* in Aizawl city. Considering the above beneficial attributes superior in terms of sensory, physico-chemical, and most importantly the microbiological qualities of CS and DS RTE *Vawksa rep*, it would command higher market demand and price in comparison to the uncured CS and DS RTE *Vawksa rep*, because nowadays, consumers are more quality conscious and ready-to-pay more for the better quality products. Besides, production technology, up-gradation of traditional meat food products is the need of the hour as these products suit to the taste and flavor of local populates.

**Table-6:** Economics of *Vawksa rep* (smoked pork product).

Commodities used	Cured (Rs.)	Uncured (Rs.)
Pork (25 kg)	6250.00	6250.00
Salt and sugar	26.25	-
Nitrate and nitrite	17.00	-
Spice mix	150.00	-
Yoghurt	100.00	-
STPP	93.00	-
Packaging	20.00	20.00
Sawdust/wood	20.00	20.00
Electricity	63.00	63.00
Total cost (25 kg)	6739.25	6353.00
Cost/kg	269.57	254.12

STPP=Sodium tripolyphosphate

## Conclusion

From the above study, it can be concluded that using combination of curing, cooking, and smoking, a new and quality assured RTE *Vawksa rep* could be developed. Compared to uncured CS and DS (T-1 and T-2), the cured CS and DS (T-3 and T-4) RTE *Vawksa rep* registered superior physico-chemical properties (higher cooking yield, favorable pH, lower TV, and lower TBA values), sensory properties (higher odor scores, higher color, flavor, texture, juiciness, and overall acceptability scores), and microbiological properties (lower TPC, absence of colititer, and *E. coli* count) throughout the storage period of 15-day. Furthermore, cured CS and DS RTE *Vawksa rep* could be prepared easily with little technology up-gradation and with a negligible escalation of production cost.

## Authors’ Contributions

The present study was a part of DD’s original research work during his M.V.Sc. thesis program. PH helped in designing of the work and gave the guidelines during experimental study. LC assisted in the processing of samples. TPS, PS, and ST assisted in statistical analysis, interpretation of the results and drafting of the manuscript. All the authors have read and approved the final manuscript.
